# Supplementary material for: Association between oral microbiome and seven types of cancers in East Asian population: a two-sample Mendelian randomization analysis
Source: Front Mol Biosci. 2023 Nov 21;10:1327893. doi: 10.3389/fmolb.2023.1327893 (PMC10702768; doi:10.3389/fmolb.2023.1327893)
Supplement: Supplementary file 1 [file DataSheet1.ZIP › Supplementary Materials/MR plots of saliva/saliva═╝/Breast cancer/pheno.347_to_breast cancer_leave_one_out.pdf]

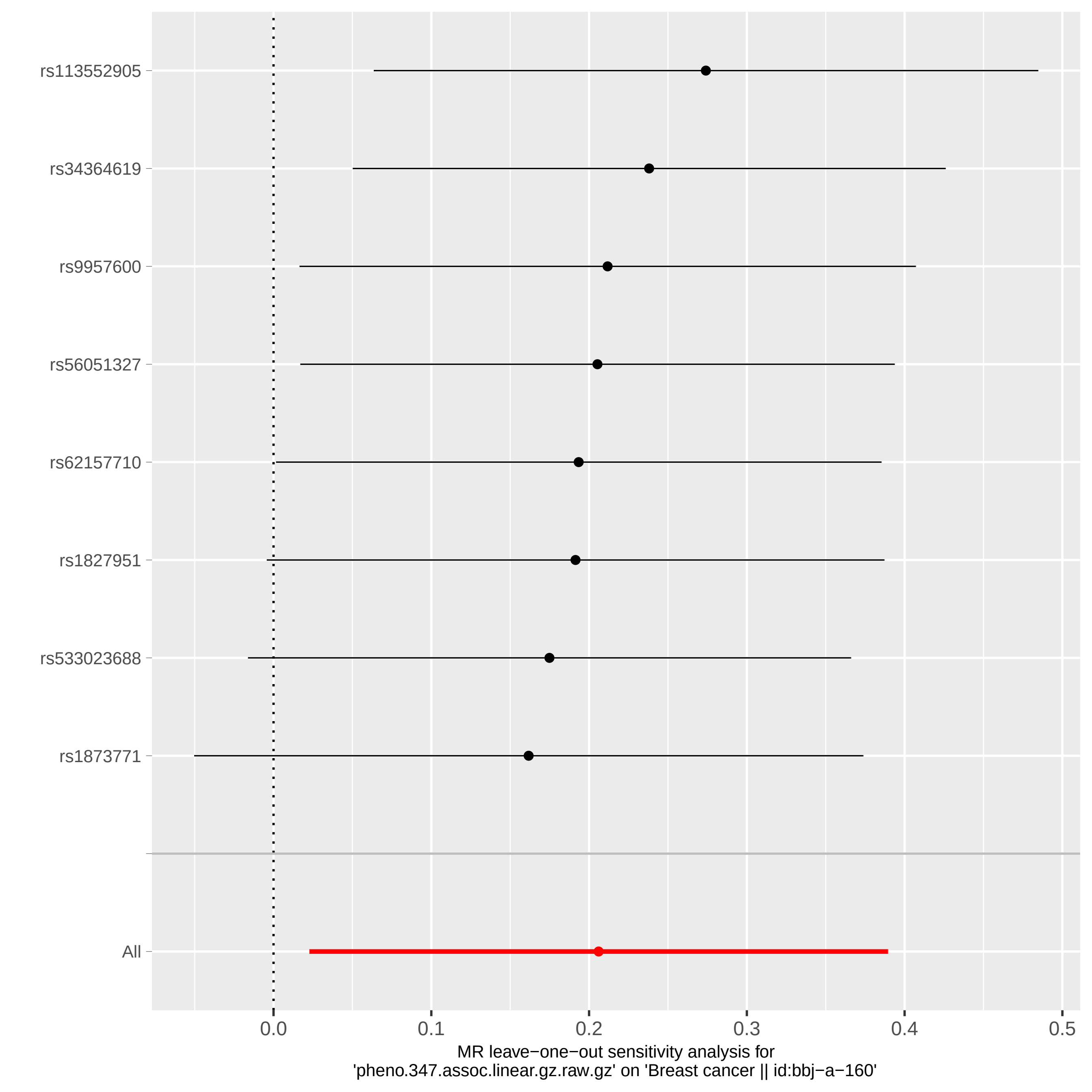

rs113552905
rs34364619
rs9957600
rs56051327
rs62157710
rs1827951
rs533023688
rs1873771
All
0.0
0.1
0.2
0.3
0.4
0.5
MR leave−one−out sensitivity analysis for
'pheno.347.assoc.linear.gz.raw.gz' on 'Breast cancer || id:bbj−a−160'
